# Effects of fotemustine or dacarbasine on a melanoma cell line pretreated with therapeutic proton irradiation

**DOI:** 10.1186/1756-9966-28-50

**Published:** 2009-04-09

**Authors:** Aleksandra M Ristić-Fira, Lela B Korićanac, Jelena J Žakula, Lucia M Valastro, Gioacchin Iannolo, Giuseppe Privitera, Giacomo Cuttone, Ivan M Petrović

**Affiliations:** 1 Vinča Institute of Nuclear Sciences, PO Box 522, 11001 Belgrade, Serbia; 2 INFN Laboratori Nazionali del Sud, via S. Sofia 62, 95123 Catania, Italy; 3 Istituto Oncologico del Mediteraneo, via Penninazzo 7, 95029 Viagrande, Italy; 4 Institut of Radiology and Radiation Oncology, University of Catania, via S. Sofia 78, 95123 Catania, Italy

## Abstract

**Background:**

Considering that HTB140 melanoma cells have shown a poor response to either protons or alkylating agents, the effects of a combined use of these agents have been analysed.

**Methods:**

Cells were irradiated in the middle of the therapeutic 62 MeV proton spread out Bragg peak (SOBP). Irradiation doses were 12 or 16 Gy and are those frequently used in proton therapy. Four days after irradiation cells were treated with fotemustine (FM) or dacarbazine (DTIC). Drug concentrations were 100 and 250 μM, values close to those that produce 50% of growth inhibition. Cell viability, proliferation, survival and cell cycle distribution were assessed 7 days after irradiation that corresponds to more than six doubling times of HTB140 cells. In this way incubation periods providing the best single effects of drugs (3 days) and protons (7 days) coincided at the same time.

**Results:**

Single proton irradiations have reduced the number of cells to ~50%. FM caused stronger cell inactivation due to its high toxicity, while the effectiveness of DTIC, that was important at short term, almost vanished with the incubation of 7 days. Cellular mechanisms triggered by proton irradiation differently influenced the final effects of combined treatments. Combination of protons and FM did not improve cell inactivation level achieved by single treatments. A low efficiency of the single DTIC treatment was overcome when DTIC was introduced following proton irradiation, giving better inhibitory effects with respect to the single treatments. Most of the analysed cells were in G1/S phase, viable, active and able to replicate DNA.

**Conclusion:**

The obtained results are the consequence of a high resistance of HTB140 melanoma cells to protons and/or drugs. The inactivation level of the HTB140 human melanoma cells after protons, FM or DTIC treatments was not enhanced by their combined application.

## Background

The disseminated melanoma is generally not curable using conventional clinical tools. Despite recent advances in the immunotherapy and vaccinotherapy, the chemotherapy remains the standard therapeutic option [1]. However, the malignant melanoma frequently displays primary chemoresistance, and only a few cytotoxic drugs have shown activity against this type of tumor. Higher remission rates are obtained with the DNA-alkylating agents, including cisplatin, methylating agents such as dacarbazine and temozolomide, or chloroethylating agents such as 2-chloroethylnitrosoureas [2].

Fotemustine (FM) is a member of the chloroethylnitrosourea class of alkylating agents that has been proven active against the disseminated melanoma and primary brain tumours [3]. Spontaneous decomposition of nitrosoureas generates electrophilic species, which are responsible for DNA alkylation, thus producing therapeutic effects. The generation of isocyanates cause toxic side effect of FM which are monitored through carbamoilation of proteins [4]. The monofunctional alkylating agent dacarbazine (DTIC) is approved and frequently used for the treatment of melanoma. Relative response after DTIC treatment is observed in 15 to 20% of cases with short duration [5,6]. Due to the inherent drug-resistant characteristic of this disease, chemotherapy is an ineffective mean of treating malignant melanoma. The reasons for the chemoresistant phenotype in human melanoma are not well understood and are probably multifactorial.

Some forms of specially localized melanoma tumors, are presently treated with therapeutic proton beams giving positive results [7]. Physical properties of protons, such as their well defined range, with the small lateral scattering and high energy deposition within the Bragg peak maximum, made this type of therapy suitable for localized melanomas. In order to treat the malignant growth with protons so that the desired uniform dose can be delivered over the large volume at the given depth, the Bragg peak is spread out by the modulation of proton energy, followed by the slight increase of the entrance dose. Various authors have reported data on modulated proton beams with energy less than 100 MeV which are used for the treatment of eye melanoma [8,9].

With the goal to find a more efficient way to treat melanoma, combined treatments of either FM or DTIC with proton irradiations were examined. In our previous studies, we investigated the effects of proton irradiations and single drug treatments on HTB140 cells, as well as the effects of proton irradiations on these cells that were pre-treated with FM or DTIC [10-12]. The objective of the present study is to examine whether the change in order and duration of treatments applied have the influence on cell inactivation level. Therefore, cell viability, proliferation, survival and cell cycle distribution were investigated on HTB140 human melanoma cells that were first irradiated and than exposed to FM or DTIC.

## Methods

### Cell Culture

The human melanoma HTB140 cells were purchased from the American Tissue Culture Collection (Rockville, MD, USA). They were grown in the RPMI1640 medium supplemented with 10% fetal bovine serum, penicillin-streptomycin and L-glutamine. The cells at the passage number 35 to 60 were maintained in 6 ml of the medium in 25-cm^2 ^plastic tissue culture flasks (Nunclon™, Amex Export -Import, Serbia) at 37°C in a humidified atmosphere with 5% CO_2_. Under these conditions, the plating efficiency (PE) for the HTB140 cells was 62 ± 7.3%, while the doubling time (Td) evaluated from the growth curve was 24 ± 2.7 h.

### Irradiation Conditions

The exponentially growing cells were irradiated within the spread out Bragg peak (SOBP) of the 62 MeV proton beam at the CATANA (Centro di Adro Terapia e Applicazzioni Nucleari Avanzati) treatment facility. The applied doses were 12 or 16 Gy at the dose rate of 15 Gy/min. These are the doses commonly used in proton therapy. The irradiation position in the middle of SOBP was obtained by interposing 16.3 mm thick Perspex plate (Polymethyl methacrylate – PMMA) between the final collimator and the cell monolayer. The obtained relative dose was 99.42 ± 0.58%, having the mean energy of protons of 34.88 ± 2.15 MeV. The reference dosimetry was performed using plane-parallel PTW 34045 Markus ionization chamber which was calibrated according to the IAEA code of practice [13,14]. All irradiations were carried out in air at room temperature. Described irradiation conditions were the same for single irradiations and combined treatments of irradiation and drugs. The biological assays that follow were performed 7 days after each irradiation.

### Chemotherapeutic Drug Treatments

The chemotherapeutic drugs used were fotemustine (FM, Ital Farmaco S.p.A., Milano, Italy) or dacarbazine (DTIC, Aventis Pharma S.p.A., Milano, Italy). Stock solutions of the drugs made for this study were prepared according to the manufacturer's instructions: 10 mM FM diluted in 43.3% ethanol and 10 mM DTIC diluted in water.

In a previous study a wide range of FM or DTIC concentrations and incubation periods were investigated [10]. It has been shown that the concentrations of 100 and 250 μM, after the incubation period of three days, produced the cell inactivation level of about 50%. Based on the obtained results, in the experimental setup described here, these values were used as relevant for the single drug and the combined radiation and drug effects.

For the single drug treatments cells were seeded at a suitable number into 25-cm^2 ^plastic tissue culture flasks or on 96-well plates, depending on the biological assay to be used. After 24 h the cells were treated with drugs (100 or 250 μM) without replating and all biological assays were performed 72 h later.

In the treatment combining proton irradiation and drugs, after being irradiated exponentially growing cells were detached by trypsinization (1.98% trypsin/0.02% EDTA in PBS), replated appropriately for each biological assay and incubated for 4 days under standard conditions (37°C, 5% CO_2_). Then the culture medium was replaced with the fresh medium containing drugs (100 or 250 μM) and the cells were incubated for additional 72 h. In this way the biological assays were carried out after the incubation period of 7 days after irradiation.

### Viability Assay

The sulforhodamine B (SRB) assay, which is based on the measurement of cellular protein content, was used for the determination of cell density. The assay was performed according to the method of Skehan and co-workers [15]. After incubation, the cells that were grown in 96-well plates (four wells per dose or concentration in each of three independent experiments) were fixed with 10% trichloroacetic acid and stained for 30 min, when the excess dye was removed by washing with 1% acetic acid. The protein-bound dye was dissolved in 10 mM tris base solution for the determination of absorbance at 550 nm using a microplate reader (Victor, Wallac).

### Proliferation Assay

The DNA synthesis and cell proliferation were measured using a 5-bromo-2-deoxyuridine (BrdU) assay (Roche Diagnostics GmbH, Mannheim, Germany). The cells were grown in 96-well plates (four wells per dose or concentration in each of three independent experiments) and BrdU labeling was performed according to the manufacturer's instructions. The absorbance was measured at 550 nm using a microplate reader (Victor, Wallac).

### Clonogenic Assay

After irradiation or drug treatment the cells were harvested by the trypsinization, seeded into 25-cm^2 ^plastic tissue culture flasks (four flasks per dose or concentration in each of three independent experiments) at a suitable number for colony assay and incubated at 37°C for 7 days. This incubation period is appropriate since it represents more than six cell-doubling times. Moreover, the results of the colony assay that was performed 14 days after irradiation did not show statistically significant differences in the cell inactivation level with respect to those obtained after 7 days [16]. Therefore, in the combined treatments, during post irradiation incubation, the drugs were introduced after 4 days (without replating), and the cells were further incubated for 3 days. The cells were then fixed with methanol and stained with 10% Giemsa solution for the evaluation of the survival.

### Flow cytometry

The cells were grown in 25-cm^2 ^plastic tissue culture flasks (four flasks per dose or concentration in each of two independent experiments). For the flow cytometric evaluation of the cell cycle status 1 × 10^6 ^cells were taken from each flask, washed with Phosphate Buffered Saline (PBS), fixed overnight with 70% cold ethanol and stained with PBS buffer that contained 50 μg/ml Propidium Iodide (PI) and 50 μg/ml RNase. After the incubation for 30 min at room temperature, the cells were analyzed by the flow cytometry (Coulter EPICS XL; Beckman Coulter) using the XL SYSTEM II software.

### Statistical analysis

Quadruplicate measurements were made during each experiment, while each experiment has been repeated three times, except for flow cytometry that was performed in two replicate experiments. All obtained data for viability, proliferation and survival assays were normalized to the untreated controls to obtain percentage of cells or surviving fraction. The significance of differences among the experimental groups was assessed by the independent Student's *t*-test, with the level of significance set at p < 0.05. Results were presented as the Mean ± S.D. (standard deviation). All data processing was carried out using the software OriginPro 7.5.

## Results

### The effects of protons and FM on cell viability, proliferation and survival

**Figure 1:**
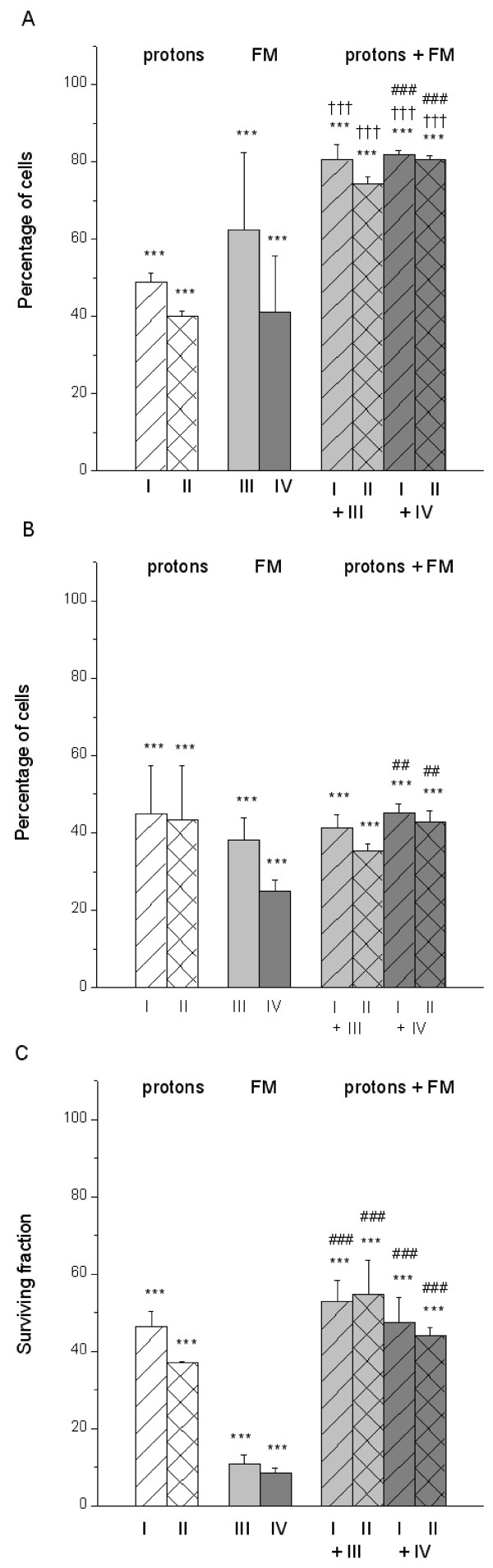
**Single and combined effects of protons and FM on HTB140 cells**. Viability (A), proliferation (B) and survival (C) of HTB140 cells estimated by SRB, BrdU and clonogenic assays, respectively, after single and combined treatments with protons and FM. Irradiation doses were 12 (I) and 16 Gy (II). Drug concentrations were 100 (III) and 250 μM (IV). (* – single or combined treatment vs. control, † – combined treatment vs. proton irradiation, # combined treatment vs. single drug treatment; 0.01 < p < 0.05 (*, †, #), 0.001 < p < 0.01 (**, ††, ##), p < 0.001 (***, †††, ###)).

Single treatments with protons or FM, presented in Figure 1A and Figure 1B, have shown dose or concentration dependent inhibitory effects on cell viability and cell proliferation, respectively, as compared to untreated controls (***, p < 0.001).

After combined treatments with these agents, as compared to controls, cell viability also decreased (***, p < 0.001) and is shown in Figure 1A. But, the single effects of either proton irradiation or FM treatment were better than those of their combined application (†††, p < 0.001 and ###, p < 0.001).

Cell proliferation after combined treatments, given in Figure 1B, was significantly reduced compared to untreated cells (***, p < 0.001). Combined effects of protons and 100 μM FM remained in the range that was obtained for each single treatment (p > 0.05). Still, cell proliferation after single treatment with 250 μM FM was lower than after its combination with protons (##, p < 0.01).

Cell survival, estimated through the colony forming ability, revealed important reduction for single and combined treatments *vs*. control (***, p < 0.001), as shown in Figure 1C. Combined effects of protons and FM were in the range of those of proton irradiation (p > 0.05) and did not reach the level of cell killing obtained by FM alone (###, p < 0.001).

### The effects of protons and DTIC on cell viability, proliferation and survival

**Figure 2:**
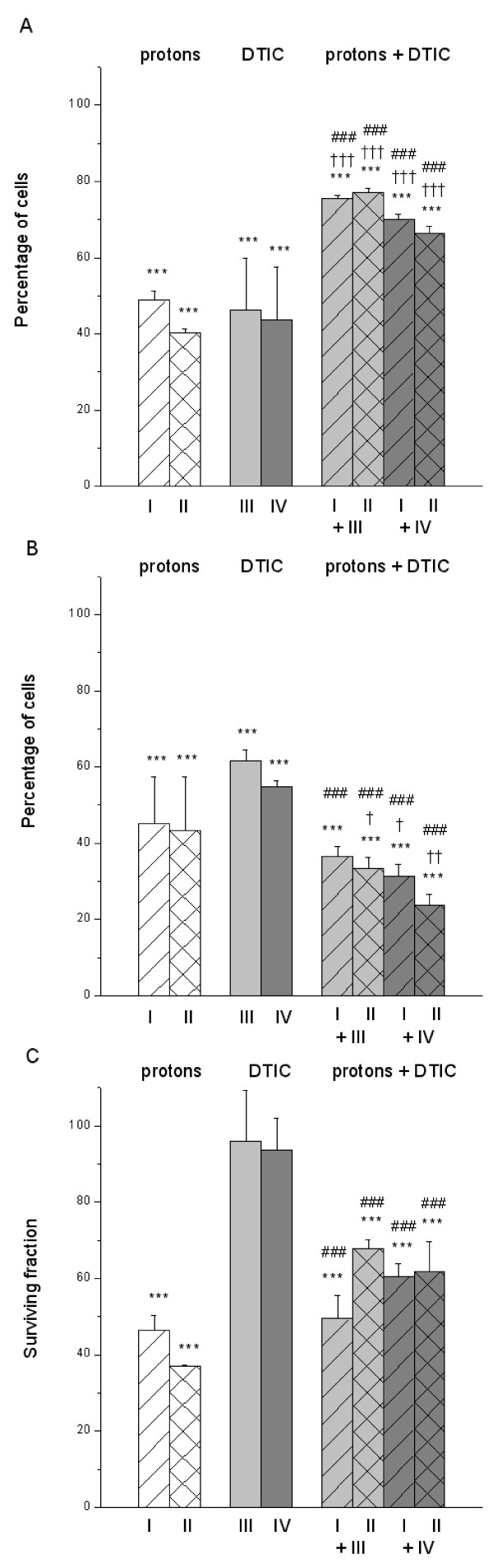
**Single and combined effects of protons and DTIC on HTB140 cells**. Viability (A), proliferation (B) and survival (C) of HTB140 cells estimated by SRB, BrdU and clonogenic assays, respectively, after single and combined treatments with protons and DTIC. Irradiation doses were 12 (I) and 16 Gy (II). Drug concentrations were 100 (III) and 250 μM (IV). (* – single or combined treatment vs. control, † – combined treatment vs. proton irradiation, # combined treatment vs. single drug treatment; 0.01 < p < 0.05 (*, †, #), 0.001 < p < 0.01 (**, ††, ##), p < 0.001 (***, †††, ###)).

After exposure to single and combined treatments with protons and DTIC, as shown in Figure 2A, the viability of HTB140 cells was reduced as compared to controls (***, p < 0.001). However, the effects of single proton irradiation or DTIC treatment were more pronounced than their combination (†††, p < 0.001 and ###, p < 0.001).

There was a high inhibition of cell proliferation after single and combined treatments with protons and DTIC, as compared to control cells (***, p < 0.001), and is given in Figure 2B. The effects of combined treatments were stronger than those of relevant single treatments, particularly regarding DTIC (†, p < 0.05; ††, p < 0.01 and ###, p < 0.001).

A reduction of cell survival vs. control, as it is shown in Figure 2C, was obtained after single proton irradiation or combination of protons and DTIC (***, p < 0.001) and was in the same range. Single DTIC treatment provoked negligible cell inactivation.

### The effects of protons and FM or DTIC on cell cycle distribution

**Figure 3:**
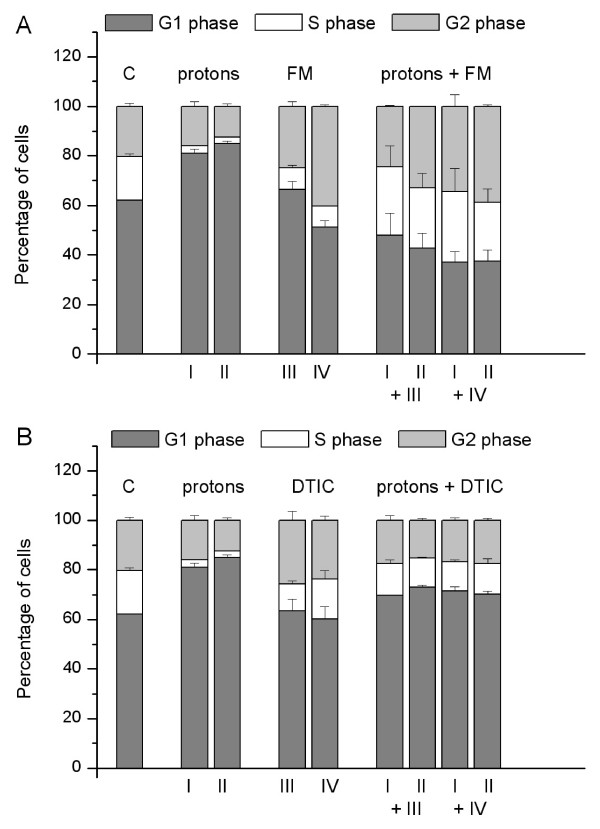
**Cell cycle analyses after single and combined treatments**. Cell cycle analysis of HTB140 cells estimated by flow cytometry, after single and combined treatments with protons and FM (A) or protons and DTIC (B). Irradiation doses were 12 (I) and 16 (II) Gy, while drug concentrations were 100 (III) and 250 μM (IV). The percentage of cells in G_0_/G_1_, S and G_2_/M phase were obtained with the XL SYSTEM II software.

Compared to untreated controls, proton irradiation of HTB140 cells induced a dose dependent increase of G1 cell population. FM provoked a raise of G2 phase followed by a reduction of S phase with some changes in G0/G1 cell population. After combined treatments with protons and FM, there was an improvement of S and G2 phase followed by a decrease of G0/G1 cell population (Figure 3A). It appears that the major characteristic of combined treatment with respect to single protons or FM was an increase of S phase mostly compensated by a reduction of G0/G1 phase.

Single DTIC treatment did not provoke changes in the cell cycle distribution as compared to control. It differed from proton effects by an increase in S and G2 cell population. Cell cycle distribution after combined application of protons and DTIC remained in the range of controls and single DTIC effects (Figure 3B).

## Discussion

Radio- and chemoresistance of malignant melanoma can be related to the phenotypic heterogeneity, including different degrees of cellular pigmentation, diverse cell morphology and growth rate of variety of melanoma types [17,18]. It has been shown that when using conventional radiation, the common radiosensitivity parameter, the surviving fraction at 2 Gy of different melanoma cell lines ranged from 0.36 to 0.96 [16,19,20]. The HTB140 human melanoma cells are among cell lines with the highest values, thus representing the limit case of cellular radioresistance. To increase the inactivation level these cells were irradiated with protons that have higher linear energy transfer than conventional radiation. Still, the surviving fraction at 2 Gy remained high with the value of 0.93 [16].

Response of the HTB140 cells to different chemotherapeutic drugs is uneven and corresponds to moderate cellular inactivation [10]. Although FM and DTIC are the members of the alkylating agent family, they inactivate HTB140 cells in different ways. Due to its major inherent toxicity, FM exhibited very high killing ability within the incubation time proper for the evaluation of clonogenic survival, i.e. 7 days after administration [10]. The highest effectiveness of the single DTIC treatment was 72 h after its administration and it almost disappeared with the prolonged incubation up to 7 days [10]. Therefore, as an example of cellular resistance, the human HTB140 melanoma cell line was used as a model system.

To achieve better cellular inactivation than it has been obtained by single treatments with either protons or drugs a study of combined effects of these agents has been undertaken. Irradiation doses were those frequently used in proton therapy [16], whereas drug concentrations were close to those that produce 50% of growth inhibition [3,10,21].

The level of cellular radiosensitivity is almost exclusively assessed by clonogenic assay. Different viability tests, for instance SRB, microtetrasolium (MTT) or BrdU are basically employed for the estimation of cellular chemosensitivity. They are also adapted for the evaluation of the cellular response to the radiation damage [22,23]. All biological assays used in this study were selected to enable the comparison of sensitivity levels of HTB140 cells after applying radiation, alkylating agents or their combination. These methods were particularly chosen because they measure distinct biological parameters in cells [24-26].

In combined treatments the common order of administration of different agents is exposure to drug and then to radiation [27,28]. Consequently, in an initial experiment the HTB140 cells were pretreated with FM or DTIC (100 or 250 μM) and were irradiated with protons (12 or 16 Gy) 24 h later [11]. Cell viability was assessed 48 h after irradiation, the time appropriate to the maximum drug effect [10,21]. For all treatments the obtained levels of viability were about 50%, without major changes between single and combined applications. The viability levels in these combined treatments are probably due only to the effects of drugs [11].

In another experimental setup the effects of combination of drugs and protons were estimated 7 days after irradiation of HTB140 cells [12]. The selected time point is proper for the evaluation of radiobiological survival, i.e., survival after at least six doubling times following irradiation. This combination of FM and protons considerably reduced cell proliferation, providing better inactivation level than each single treatment. Effects of the combination of DTIC and protons were small for cell proliferation and viability [12].

According to the discussed results of the two experiments, an improvement of combined treatments, with respect to the single once, was achieved only after the combination of FM and protons, 7 days after irradiation [11,12].

To increase the efficiency of combined treatments, particularly the combination of DTIC and protons, the order of administration of drugs and radiation was inversed. The new experimental set up was conceived knowing the position on the time scale where the best effect of each single treatment with FM, DTIC or protons was reached [10]. The HTB140 cells were irradiated with protons, incubated for 4 days, when FM or DTIC was added to the cells, and then incubated for another 3 days. In this way it was enabled that the incubation periods providing the best single effects of protons and drugs coincide at the same time.

The described combination of protons and FM reduced cell proliferation to ~40% and clonogenic survival to ~50%, while there was ~80% of viable cells estimated by the SRB assay (Figure 1). With respect to the single treatments the obtained effects were weaker. The time interval between irradiation and drug treatment might be considered as long because the multiplicity of microcolonies 4 days after irradiation could underestimate the effects of drug treatment, particularly for the clonogenic assay. An overestimation of cell viability by the SRB assay could be ascribed to the excess of proteins coming from the dead cells that were indistinguishable from those of surviving cells [23]. DNA damaging agents also produce morphological changes of cells, such as an increased cell size and therefore protein content [29]. This might also explain the overestimated viability obtained by the SRB assay. Comparing the inactivation levels obtained in this experiment to those of the two experiments that were previously described [11,12], the best effect was obtained when the HTB140 cells were treated with FM before proton irradiation and incubated for 7 days [12].

The combination of protons and DTIC reduced cell proliferation to ~32% while after single treatments this level was higher (Figure 2). Again, an overestimation of viability was obtained by SRB assay [23,29]. According to cell proliferation and survival, the poor efficiency of the single DTIC treatment was overcome when it was introduced following proton irradiation. The cells that were damaged by protons and would most likely survive were additionally damaged in a similar way by the DTIC treatment [30]. As a result, the obtained cell inactivation levels were better than those of the two previously reported experiments [11,12].

Analysing the effects of the two administration procedures of radiation and drugs, in general there was not an appreciable improvement with respect to the single treatments. In each of them there was a moderate improvement with the combination of just one drug and radiation.

All studied agents affect cellular DNA, but they differ in the type of damage they induce. Protons, as well as conventional radiation, induce oxidative changes in DNA bases together with the single- and double-strand breaks [31]. Alkylating agents act by transferring methyl, ethyl or chloroethyl group to DNA [32]. The drugs of nitrosourea type, such as FM, express high cytotoxicity through the formation of interstrand cross-links in DNA [33].

The dominating mechanism of chemoresistance to alkylating agents is the repair of DNA adducts by the enzyme O^6^-methylguanine DNA-methyltransferase [3]. Ionizing radiation also induces activity of this enzyme [34]. In melanoma cells exposed to the alkylating agents or ionizing radiation the level of O^6^-methylguanine DNA-methyltransferase may increase, resulting in a resistance to such treatments. Some melanoma cell lines inherently express high level of O^6^-methylguanine DNA-methyltransferase [5]. The weak effect of combined treatments is due to the relatively high level of O^6^-methylguanine DNA-methyltransferase that might be intrinsically present in the HTB140 cells and/or triggered by proton irradiation.

Another possible reason for such a limited effectiveness of the combination of protons and drugs is the nuclear transcription factor kappa B (NF-κB) that is constitutively expressed in melanoma cells [35]. NF-κB is an important feature in the development and progression of malignancies by targeting genes that promote cell proliferation, survival, metastasis and angiogenesis. NF-κB also regulates apoptosis by controlling the transcription of genes that block cell death. Activation of NF-κB induces overexpression of bcl-x_l_, bcl-2, vascular endothelial growth factor and interleukin-8. This may affect resistance to apoptosis induced by radiation and chemotherapy [36]. Alkylating agents as well as ionizing radiation can induce cell death through the activation of apoptosis [21,28,37]. However, the described mechanism can cause defects in apoptotic pathways, leading to a high cellular resistance [35].

In the HTB140 cells proton irradiation induced G1 phase arrest, while FM as well as combined treatments provoked significant G2 arrest (Figure 3A). After ionizing radiation a delay in G2 phase is the most frequent event, but significant delays could also occur in G1 and S phase [38]. These results are in agreement with the high radioresistance of HTB140 cells [16]. FM generally produces a G2/M block in the cell cycle, while higher drug concentrations could induce S phase accumulation [39]. In samples exposed to FM or in combined treatments the cell proliferation (Figure 1B) was in agreement with the S phase (Figure 3A). Combined treatment with protons and DTIC, did not induce major changes in the cell cycle as compared to the control or single DTIC treatment (Figure 3B). Similar cell cycle arrest in S and G2/M phase caused by DTIC was also reported for other melanoma cells [40]. Compared to protons, after combined treatment there was a slight reduction of G1 phase and an increase of S phase. Most of the analysed cells were in G1/S phase, thus being viable and able to replicate DNA.

The obtained FACS results may be influenced by a specific feature of melanoma cells which is melanogenesis. This metabolic activity of melanoma cells triggers arrest and accumulation of cells in the G1 phase [41]. FACS analyses of the HTB140 cells did not show a major accumulation of cells in G2/M phase 7 days after irradiation, confirming that these cells are among very radioresistant lines, as it was already reported for the viability and survival [16].

## Conclusion

To improve single effects of protons, FM or DTIC on the inactivation of HTB140 melanoma cells, combined treatments with these agents have been investigated. After being irradiated with protons cells were exposed to either FM or DTIC. The combination of protons and FM did not improve the cell inactivation level achieved by each single treatment. The poor efficiency of the single DTIC treatment was overcome when DTIC was introduced following proton irradiation, giving better inhibitory effects with respect to the single treatments. The molecular mechanisms activated by protons enabled DTIC to express its cytostatic nature. However, under the studied experimental conditions the level of sensitivity of the HTB140 cells to protons, FM or DTIC remained within 50% of cell inactivation also after their combined application.

## Competing interests

The authors declare that they have no competing interests.

## Authors' contributions

AMRF, IMP, GC and GP designed the experiments. LBK and JJŽ carried out cell culture experiments and viability tests. GI performed FACS analysis. AMRF, IMP, LMV and GC carried out the irradiation experiments. LBK performed the statistic analysis. AMRF and IMP supervised the experiments and drafted the manuscript. All authors have read and approved the final version of the manuscript.
